# Multilocus perspectives on the monophyly and phylogeny of the order Charadriiformes (Aves)

**DOI:** 10.1186/1471-2148-7-35

**Published:** 2007-03-08

**Authors:** Matthew G Fain, Peter Houde

**Affiliations:** 1 Department of Biology, New Mexico State University, Box 30001 MSC 3AF, Las Cruces New Mexico 88003 USA

## Abstract

**Background:**

The phylogeny of shorebirds (Aves: Charadriiformes) and their putative sister groups was reconstructed using approximately 5 kilobases of data from three nuclear loci and two mitochondrial genes, and compared to that based on two other nuclear loci.

**Results:**

Charadriiformes represent a monophyletic group that consists of three monophyletic suborders Lari (i.e., Laridae [including Sternidae and Rynchopidae], Stercorariidae, Alcidae, Glareolidae, Dromadidae, and Turnicidae), Scolopaci (i.e., Scolopacidae [including Phalaropidae], Jacanidae, Rostratulidae, Thinocoridae, Pedionomidae), and Charadrii (i.e., Burhinidae, Chionididae, Charadriidae, Haematopodidae, Recurvirostridae, and presumably Ibidorhynchidae). The position of purported "gruiform" buttonquails within Charadriiformes is confirmed. Skimmers are most likely sister to terns alone, and plovers may be paraphyletic with respect to oystercatchers and stilts. The Egyptian Plover is not a member of the Glareolidae, but is instead relatively basal among Charadrii. None of the putative sisters of Charadriiformes were recovered as such.

**Conclusion:**

Hypotheses of non-monophyly and sister relationships of shorebirds are tested by multilocus analysis. The monophyly of and interfamilial relationships among shorebirds are confirmed and refined. Lineage-specific differences in evolutionary rates are more consistent across loci in shorebirds than other birds and may contribute to the congruence of locus-specific phylogenetic estimates in shorebirds.

## Background

The order Charadriiformes is one of relatively few examples in which the phylogenetic relationships of a major higher-level clade of birds are becoming successfully resolved [1,2]. The order includes what have traditionally been known as the shorebirds, a diverse and apparently ancient group of non-passerine birds whose three suborders are estimated to have diverged from one another in the Cretaceous [3]. Earlier morphological and biochemical analyses produced conflicting pictures of shorebird phylogeny. Morphological and biochemical studies were in general agreement as to recognition of the suborders Charadrii, Scolopaci, and Lari as clades. However, morphological studies also recognized the Alci as distinct [4,5], whereas DNA-DNA hybridization placed them near gull-like birds in the Lari. In contrast, recent molecular studies sampling both nuclear and mitochondrial sequences have generated a remarkably consistent and highly-resolved interfamilial tree for Charadriiformes [2,3,6,7].

While the monophyly of Charadriiformes is popularly accepted, it has been questioned both implicitly and explicitly. Olson and Feduccia hypothesized that charadriiform stilts (i.e., Recurvirostridae) were ancestral to both waterfowl (Anseriformes) and flamingos (Phoenicopteridae) based on their interpretation of fossils and comparative anatomy [8-11]. Like some early anatomists of the 19^th ^century, Olson portrayed the "gruiform" bustards (Otididae) as Charadriiformes, closely related to the coursers (Glareolidae) and in particular to the Egyptian Plover [10]. He further advocated an ill-defined relationship of ibises (Ciconiiformes: Threskiornithidae) "uniting" Gruiformes and Charadriiformes [10,12]. Sibley et al. found rails (Rallidae) to be statistically inseparable from both Gruiformes and Charadriiformes using DNA-DNA hybridization [13].

These hypotheses have been discredited piecemeal in recent years. Specifically, there exists strong evidence for the sistership of waterfowl and fowl as Galloanserae, the sister to Neoaves [14-16]. There is also strong evidence for a clade of flamingos and grebes [17,18] within Metaves, one of two hypothesized basal clades of Neoaves (the other being Coronaves, to which shorebirds belong) [7,19]. All subsequent studies have upheld the novel transfer of both Australian Plains-wanderer (Pedionomidae) and buttonquails (Turnicidae) from the order Gruiformes to Charadriiformes [2,3,7,20,21].

However, many of the proposed interrelationships of gruiform, charadriiform, and ciconiiform taxa have not yet been explicitly tested in a comprehensive molecular phylogenetic framework with both evidence from multiple independent loci and comprehensive taxon sampling. Gruiformes or sandgrouse (Pterocliformes) have been cited most commonly as potential sister groups of Charadriiformes and representative members of these groups have generally been used to root a presumed monophyletic Charadriiformes. The monophyly of Charadriiformes has been tested only with limited taxon sampling [6]; with incomplete DNA-DNA hybridization matrices [21]; or with single-locus studies [7]. In the process of studying the phylogenetic relationships of Gruiformes [22]we had the opportunity to characterize and analyze more than 5 kb of DNA sequences from four loci (mitochondrial and three nuclear) from a variety of putative sister groups of Gruiformes, including multiple representatives of most families of Charadriiformes. Our novel data include intronic and exonic sequences from *beta-fibrinogen*, *alcohol dehydrogenase-1*, and *glyceraldehyde-3-phosphate dehydrogenase*. These map to chromosomes 4 (position 20,917K), 4 (position 60,497K), and 1 (position 71,016.5K), respectively, in chicken. We present the results of phylogenetic analyses of these loci for Charadriiformes and compare our results to those of others, who independently studied the relationships of Charadriiformes using DNA sequences from two other nuclear loci (*myoglobin *chromosome 1, position 48,720.4K, and *RAG-1 *chromosome 5, position 16,597.6K in chicken) and nearly complete mitochondrial genomes [2,3,6]. We also test the monophyly and sister relationships of Charadriiformes by multi-locus sequence analysis including all the aforementioned putative ingroups or sister groups.

Of note, no single molecular phylogenetic analysis of Charadriiformes has yet included representatives of all its member families. The monotypic Ibisbill (Ibidorhynchidae) has yet to be studied by anyone, although it is generally presumed to fall within the Charadrii, near stilts. Paton et al. [3] and Paton & Baker [2] lacked DNA sequences of Ibisbill and the monotypic Crab Plover (Dromadidae). Ericson et al. [6] lacked these as well as buttonquails (Turnicidae) and the monotypic Australian Plains-wanderer (Pedionomidae). Thomas et al. [23]lacked eight of the traditionally recognized charadriiform families in their study of mitochondrial *cytochrome-b *DNA sequences. Likewise, we were unable to include Ibisbill, Crab-Plover, sheathbills (Chionididae), and the genus *Pluvianellus*, the last of which is not a member of the family Charadriidae in which it is traditionally included [3]. Our results corroborate numerous novel family-level relationships reported in the aforementioned recent studies, including the positions of the traditional gruiform Turnicidae basal to a clade of glareolids, larids, alcids and relatives (suborder Lari). In the present study, we include two putative representatives of Glareolidae, the Double-banded Courser (*Rhinoptilus africanus*) and the Egyptian Plover (*Pluvianus aegyptius*). *Pluvianus *has not been included previously in molecular analyses, and in contrast to traditional classifications, we conclude that this genus is distinct from the Glareolidae, with closer relations within the Charadrii than the Lari.

Last, some interfamilial relationships have not been well-resolved in previous single-locus nuclear or mitochondrial molecular data sets. For example, *myoglobin intron 2 *and *RAG-1 *suggested that Recurvirostridae and Haematopodidae may be nested within Charadriidae, rendering the latter paraphyletic. Also unresolved is whether skimmers ("Rynchopidae") are sister to either gulls (Larinae) or terns (Sterninae) or both [2,3,6]. Further, a potential conflict in topology exists between *myo-2 *and *RAG-1 *[6] as to whether Jacanidae is sister to Thinocoridae (seedsnipes) or to Rostratulidae (painted-snipes). The data at hand address these relationships.

## Results

### Molecular Characterization

We sampled four independent, presumably unlinked loci to test recent novel hypotheses of relationships within Charadriiformes and to test monophyly of the group comprehensively, particularly given our interest in proposed interrelationships between shorebirds, various families traditionally recognized as Gruiformes, and other groups. For taxa sampled see Table 1. Having a variety of loci with differing substitution rates and evolutionary properties is particularly desirable in a case such as this, wherein the relationships which interest us potentially span 60 to 80 million years. These include potentially relatively recent divergences between shorebird sister families to divergences between orders in the late Cretaceous [3]. Hence our sampling includes relatively rapidly-evolving mitochondrial loci; nuclear introns which have proven useful within a number of vertebrate clades including mammals, birds, and snakes; and relatively constrained nuclear exons, which may change slowly enough to retain some phylogenetic signal deep within the tree.

**Table 1:** Taxon sampling and GenBank accession numbers for loci

		GenBank accession numbers				
		
Higher Taxon	Species	*12S+Val*	*16S*	*FGB7*	*ADH5*	*GPD3-5*
Galliformes	*Callipepla gambelii*	[DQ485791]	[DQ485829]	[DQ494145]	[DQ485865]	[DQ485912]
Anseriformes	*Anseranas semipalmata*	[DQ674553]	[DQ674593]	[AY695132]	[DQ485866]	[DQ485913]
Phoenicopteriformes	*Phoenicopterus ruber*	[DQ674554]	[DQ674594]	[AY695139]	[DQ674631]	[DQ674666]
Podicipediformes	*Aechmophorus sp*	[DQ674555]	N/A	[AY695146]	N/A	[DQ674667]
	*Podylimbus podiceps*	[DQ674556]	[DQ674595]	[AY695145]	[DQ674632]	[DQ674668]
Pterocliformes	*Pterocles bicintus*	[DQ674558]	[DQ674597]	[AY695147]	[DQ674634]	[DQ674670]
	*Syrrhaptes paradoxus*	[DQ674559]	[DQ674598]	[AY695148]	[DQ674635]	[DQ674671]
Mesitornithiformes	*Mesitornis unicolor*	[DQ674557]	[DQ674596]	[AY695144]	[DQ674633]	[DQ674669]
Otidiformes	*Afrotis afra*	[DQ674591]	[DQ674629]	[AY695149]	[DQ674664]	[DQ674699]
	*Ardeotis kori*	[DQ674590]	[DQ674628]	[AY695150]	[DQ674663]	[DQ674698]
	*Eupodotis senegalensis*	[DQ674592]	[DQ674630]	[AY695152]	[DQ674665]	[DQ674700]
	*Tetrax tetrax*	[DQ674589]	[DQ674627]	[AY695151]	[DQ674662]	[DQ674697]
Ciconiiformes	*Plegadis chihi*	[DQ674561]	N/A	[AY695215]	[DQ674637]	[DQ674673]
	*Ajaia ajaja*	[DQ674560]	[DQ674599]	[AY695214]	[DQ674636]	[DQ674672]
Gruiformes	*Grus canadensis*	[DQ485815]	[DQ485853]	[AY082410]	[DQ485879]	[DQ485925]
	*Fulica americana*	[DQ485827]	[DQ485863]	[AY695244]	[DQ485887]	[DQ485933]
Charadriiformes						
Burhinidae	*Burhinus bistriatus*	[DQ674587]	[DQ674625]	[AY695198]	[DQ674660]	[DQ674695]
Charadriidae	*Charadrius vociferus*	[DQ485792]	[DQ485830]	[AY695205]	[DQ48586]	[DQ485914]
	*Pluvialis dominica*	[DQ674562]	[DQ674600]	[AY695201]	[DQ674638]	[DQ674674]
	*Vanellus resplendens*	[DQ674565]	[DQ674603]	[AY695206]	[DQ674641]	[DQ674676]
Haematopodidae	*Haematopus palliatus*	[DQ674563]	[DQ674601]	[AY695204]	[DQ674639]	[DQ674675]
Recurvirostridae	*Himantopus mexicanus*	[DQ674564]	[DQ674602]	[AY695203]	[DQ674640]	[DQ485915]
	*Recurvirostra americana*	[DQ485793]	[DQ485831]	[AY695202]	[DQ485868]	N/A
Turnicidae	*Turnix varia*	[DQ674575]	[DQ674613]	[AY695197]	[DQ674649]	[DQ674685]
Glareolidae	*Rhinoptilus africanus*	[DQ674574]	[DQ674612]	[AY695196]	[DQ674648]	[DQ674684]
	*Pluvianus aegyptius*	[DQ674588]	[DQ674626]	[AY695199]	[DQ674661]	[DQ674696]
Stercorariidae	*Stercorarius pomarinus*	[DQ674573]	[DQ674611]	[AY695195]	N/A	N/A
Alcidae	*Cepphus columba*	[DQ674572]	[DQ674610]	[AY695193]	N/A	[DQ674683]
	*Uria aalge*	[DQ485794]	[DQ485832]	[AY695192]	[DQ485869]	[DQ485916]
Rynchopidae	*Rynchops niger*	[DQ674567]	[DQ674605]	[AY695191]	[DQ674643]	[DQ674678]
Laridae	*Larus atricilla*	[DQ485795]	[DQ485833]	[AY695186]	[DQ485870]	[DQ485917]
	*Larus occidentalis*	[DQ674566]	[DQ674604]	[AY695185]	[DQ674642]	[DQ674677]
	*Sterna antillarum*	[DQ674568]	[DQ674606]	[AY695190]	[DQ674644]	[DQ674679]
	*Sterna caspia*	[DQ674569]	[DQ674607]	[AY695188]	[DQ674645]	[DQ674680]
	*Sterna forsteri*	[DQ674570]	[DQ674608]	[AY695187]	[DQ674646]	[DQ674681]
	*Sterna maxima*	[DQ674571]	[DQ674609]	[AY695189]	[DQ674647]	[DQ674682]
Scolopacidae	*Actitis macularius*	[DQ674579]	[DQ674617]	[AY695182]	[DQ674653]	N/A
	*Gallinago gallinago*	[DQ674576]	[DQ674614]	N/A	[DQ674650]	[DQ674686]
	*Limosa fedoa*	[DQ674577]	[DQ674615]	[AY695180]	[DQ674651]	[DQ674687]
	*Limnodromus sp*	[DQ674578]	[DQ674616]	[AY695183]	[DQ674652]	[DQ674688]
	*Phalaropus tricolor*	[DQ674581]	[DQ674619]	[AY695184]	[DQ674655]	[DQ674690]
	*Tringa melanoleuca*	[DQ674580]	[DQ674618]	[AY695181]	[DQ674654]	[DQ674689]
Jacanidae	*Actophilornis africanus*	[DQ674582]	[DQ674620]	[AY695178]	[DQ674656]	N/A
	*Jacana spinosa*	[DQ485796]	[DQ485834]	[AY695179]	[DQ485871]	[DQ485918]
Rostratulidae	*Rostratula benghalensis*	[DQ674583]	[DQ674621]	[AY695177]	[DQ674657]	[DQ674691]
Thinocoridae	*Attagis gayi*	[DQ674584]	[DQ674622]	[AY695175]	[DQ674658]	[DQ674692]
	*Thinocorus orbignyanus*	[DQ674585]	[DQ674623]	[AY695176]	[DQ674659]	[DQ674693]
Pedionomidae	*Pedionomus torquatus*	[DQ674586]	[DQ674624]	[AY695174]	N/A	[DQ674694]

The alignment of *FGB7 *is 1457 sites in length for the 48 included taxa (Table 2); 531 sites (36%) were removed before analysis. This large percentage of sites should not be taken to indicate overall difficulty in alignment. Most of the removed sites (316 aligned positions) were due to large apomorphic insertions in seedsnipes (Thinocoridae) and Plains-wanderer (Pedionomidae). In fact, despite length differences between sequences of *Attagis*, *Thinocorus*, and *Pedionomus*, the alignment of these large insertions could be taken as further evidence of the close relationship of these two families. This intron is relatively long with a slight excess of A and T nucleotides and relatively even rates among substitution types. Also, most sites are free to vary, allowing accumulation and retention of more phylogenetic signal per site in comparison to protein-coding sequences [24,25]. These general evolutionary properties explain why *FGB7 *is becoming widely used in avian systematics and has been successfully employed for even the deepest levels of avian phylogeny [7,19,26]. The nucleotide substitution models selected for *FGB7 *were HKY+G (hLRT) or TVM+ G (AIC) (Table 3). The latter is a more general case of the former, with each of the four transversion substitution types having a different rate. We analyzed the data with the less complex HKY model to minimize variance associated with estimating the additional rate parameters. However, an analysis with the more complex model did not substantively change the result (not shown).

**Table 2:** Characteristics of loci

			mean base frequencies^1^					heterogeneity of base frequencies^2^		
					
locus	# aligned sites (# analyzed)	# PI sites^1^	A	C	G	T	%AT	Chi square	df	P value
**ADH-I intron 5 (including partial flanking exons)**										
	851 (703)	306	0.27	0.24	0.24	0.25	52	81.80	129	1.00 (NS)
**ADH-I partial exons 5,6 **(69 codons:1^st ^position 17 variable, 2^nd ^position 4 variable, 3^rd ^position 44 variable)										
	207 (207)	57	0.25	0.24	0.28	0.23	48	52.18	129	1.00 (NS)
**ADH-I intron 5**										
	644 (499)	249	0.31	0.22	0.24	0.23	54	66.21	129	1.00 (NS)
**GAPD-H exons 4–5 and introns 3–5**										
	911 (684)	316	0.23	0.20	0.33	0.24	47	104.64	129	0.94 (NS)
**GAPD-H exons 4–5 **(65 codons: 1^st ^position 5 variable, 2^nd ^position 5 variable^2^, 3^rd ^position 35 variable)										
	196 (196)	23	0.25	0.22	0.27	0.26	51	35.46	129	1.00^3 ^(NS)
**GAPD-H introns 3–5**										
	713 (486)	292	0.20	0.22	0.33	0.32	44	96.41	129	0.99 (NS)
**FGB intron 7**										
	1475 (926)	487	0.30	0.18	0.19	0.33	63	36.35	138	1.00 (NS)
**combined mtDNA (12S rDNA, Valine-tDNA, 16S rDNA)**										
	2903 (2229)	659	0.33	0.25	0.21	0.21	54	135.95	135	0.46 (NS)
**12S rDNA**										
	1074 (851)	271	0.31	0.26	0.22	0.21	52	79.23	141	1.00 (NS)
**16S rDNA**										
	1753 (1318)	375	0.34	0.24	0.20	0.22	56	94.30	135	1.00 (NS)
**valine tDNA**										
	76 (60)	15	0.36	0.21	0.19	0.24	60	59.05	135	1.00 (NS)

^1 ^number remaining after ambiguously aligned sites removed

^2 ^base composition heterogeneity based on variable sites only

^3 ^2^nd ^position P = 0.94 (NS)

**Table 3:** Models and parameters

				R- Matrix					
				
locus	model	Pinv	alpha	A-C	C-T	A-G	A-T	C-G	G-T
**ADH-I intron 5 (with partial flanking exons)**									
	TN93+G	NA	1.41	1.0	3.34	1.0	1.0	5.09	1
**GAPD-H exons 4–5 and introns 3–5**									
	TN93+I+G	0.20	1.96	1.0	5.04	1.0	1.0	7.08	1
**FGB intron 7**									
	HKY85+G	NA	5.04	1.0	4.03	1.0	1.0	4.03	1
**combined nuclear DNA**									
	TN93+G	NA	1.70	1.0	3.92	1.0	1.0	4.98	1
**combined mtDNA**									
	GTR+I+G	0.37	0.39	4.50	19.25	3.09	0.34	60.68	1
**total combined data**									
	GTR+I+G	0.10	0.55	1.21	4.89	0.80	1.01	9.55	1

*ADH5 *shares many of the desirable properties of *FGB7*, such as even base composition and relatively uniform rates among sites (Table 2). The overall alignment is 851 sites, and 148 (17%) were removed as autapomorphic insertions or ambiguously-aligned. Of the remaining alignment, 207 sites were retained from the flanking exons. No significant heterogeneity in base composition was found among lineages for either the intron or any of the codon positions. Model selection for the entire alignment chose either the Tamura-Nei model with equal nucleotide frequencies and gamma-distributed rate variation (TNef+G; hLRT) or the GTR+G model (AIC) (Table 3). In this case, we chose a "compromise" model by relaxing the assumption of equal base frequencies, because the combination of intron and exon partitions masks some nucleotide variation between them, and within exons at each of the codon positions. In practice, this makes very little difference to the analysis because of the small number of and relatively low divergence of exon sites.

The *GPD3-5 *alignment is the most heterogeneous of the nuclear sequences, consisting of introns 3, 4, and 5, and exons 4 and 5 (Table 2). The introns, in sum, consist of 713 aligned positions, and the exons are 198 bp in length. Of the total 911 aligned sites, 227 (25%) were removed prior to phylogenetic analysis. *GPD3-5 *introns were short compared to those from *ADH-I *or *fibrinogen*, and the most problematic with respect to reliable alignment. The high proportion of sites removed reflects the fact that polypyrimidine tracts near the end of the introns made up a greater proportion of total intronic length in *GPD3-5*.

**Figure 1:**
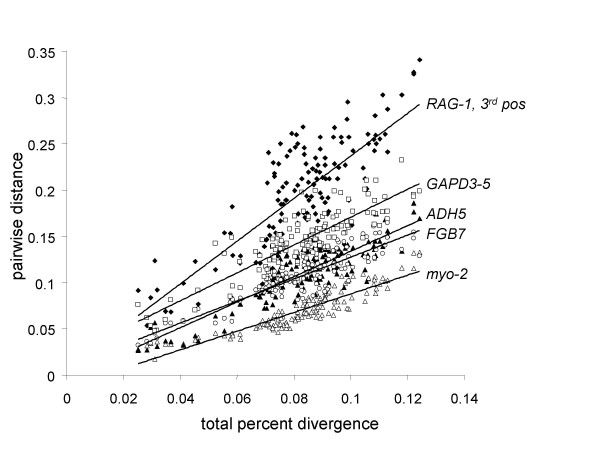
**Substitution rates per locus**. Pairwise distances of each of five non-coding partitions of nuclear loci plotted against combined pairwise distances with linear model regressions added, showing differences in evolutionary rates among loci. Closed diamonds, *RAG-1 *3^rd ^positions; open squares, *GPD3-5*; closed triangles, *ADH5*; open circles, *FGB7*; and open triangles, *myo-2*. Note faster rate of *RAG-1 *3^rd ^positions than introns.

**Figure 2:**
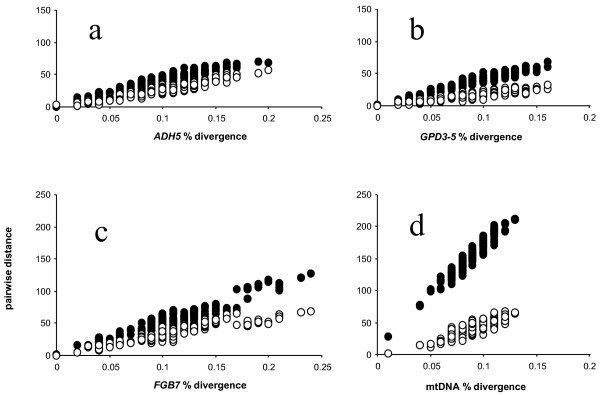
**Transition:transversion plots**. Uncorrected transition and transversion pairwise distances plotted against total distance for each of four loci obtained from combined MP analysis, drawn to same scale. (a), *ADH5*; (b), *FGB7*; (c), *GPD3-5*; and (d), *16S rDNA*, *12SrDNA*, and *tRNA Valine*. Closed circles, transition substitutions; and open circles, transversion substitutions. Note accelerated rate of transition substitutions of mtDNA.

We plotted relative rates among partitions of characters that should be nearest to the ideal of neutral substitution (Figure 1). This revealed that *GPD3-5 *intron substitutions were evolving slightly faster than the other two introns sampled in this study, while the rates of *ADH5 *and *FGB7 *were virtually identical. For this analysis, we also included the 930-bp fragment of *RAG-1 *and *myo-2 *analyzed by Ericson et al. (2003) for genera which overlapped in our study (a total of 17 taxa). Surprisingly, third positions in *RAG-1 *evolved faster than any of the introns, and *myo-2 *was actually the slowest of the introns. Not surprisingly, transition:transversion saturation plots revealed that rates of mitochondrial transitions far exceeded those of all other partitions (Figure 2).

The mitochondrial data set included genes encoding three structural RNAs, *12S rRNA*, *Valine tRNA*, and *16S rRNA *(Table 2). The total alignment of 48 taxa consisted of 2903 sites, of which 674 (23%) were excluded due to ambiguous alignment. Model selection resulted in the most complex available model in ModelTest, with six substitution types, and a proportion of invariant sites plus a gamma distribution of rate variation among sites free to vary (GTR+I+G) (Table 3). Despite the fact that *rDNA *sequences are generally considered the slowest evolving mitochondrial genes and therefore appropriate for deeper divergences, their component sites actually vary considerably in rate with 37% of sites estimated to be invariable and Chi-Square = 0.39. Typical saturation plots showed no decline in the slope of transition or transversion distances with total distance, but divergences among some ingroup taxa were greater than ingroup-outgroup comparisons. Base composition is relatively even within the *rDNA*s. For example, there is a higher percentage of G nucleotides than expected based on overall mitochondrial base composition. However, this apparent evenness masks heterogeneity among sites, particularly between stem and loop sites. When base composition is calculated only for variable sites, a more usual mitochondrial signature is seen, with an excess of A and C, and G underrepresented. This uneven composition at variable positions, combined with significant rate heterogeneity among sites and constraints on paired stem sites, suggests that at deeper levels, multiple substitutions at those sites free to vary may obscure phylogenetic signal. Nevertheless, the inferred phylogeny was largely congruent with estimates based on present and previous data sets (see below).

### Phylogenetic Reconstruction

**Figure 3:**
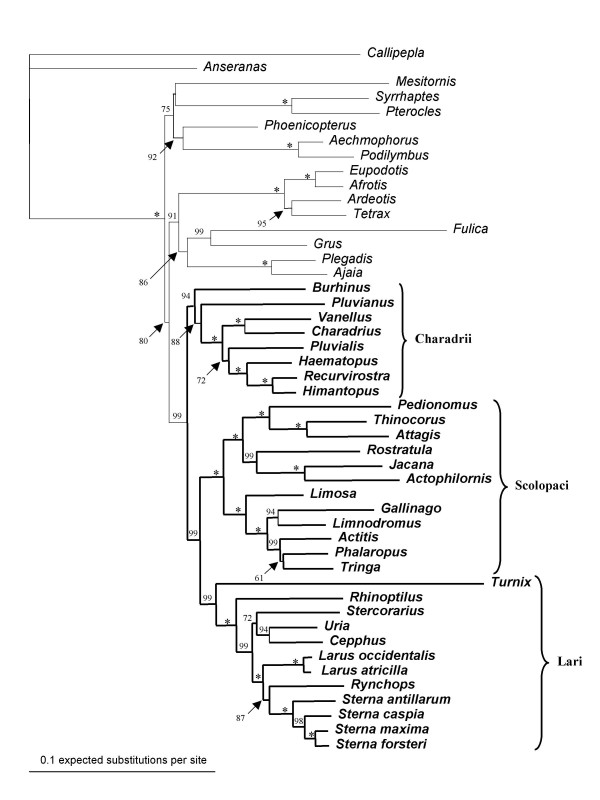
**Phylogeny of Charadriiformes**. Optimal maximum likelihood phylogenetic reconstruction of Charadriiformes and selected outgroups based on combined data of *ADH5*, *GPD3-5*, *FGB7*, *12S rDNA*, *16S rDNA*, and *tRNA Valine *using GTR + G. Both mixed model Bayesian analysis and maximum parsimony produce trees of identical topology. Bootstrap values obtained from 500 ML pseudoreplicates are indicated above branches or positioned by arrows. Asterisks indicate bootstrap values of 100%. Charadriiformes are indicated by bold font and subordinal epithets.

Maximum likelihood, mixed-model Bayesian, and maximum parsimony phylogenetic analyses of the combined data sets (Figure 3) are highly supported by bootstrap and posterior probabilities at most nodes. Relationships based on the combined data sets strongly support monophyly of Charadriiformes, and division of the order into three major clades including 1) Scolopaci (Scolopacidae, Jacanidae, Rostratulidae, Thinocoridae; and Pedionomidae) and its sister, 2) Lari (Laridae, Alcidae, Stercorariidae, Glareolidae [i.e., *Rhinoptilus*, not *Pluvianus*], and Turnicidae), and 3) their combined sister Charadrii (Burhinidae, Pluvianus, Charadriidae, Haematopodidae, and Recurvirostridae). Within Charadrii, Charadriidae (i.e., *Pluvialis*) is paraphyletic to Haematopodidae plus Recurvirostridae. Within Scolopaci, Rostratulidae and Jacanidae are sisters, and *Phalaropus *is sister to *Tringa *among those genera studied within Scolopacidae. These data provide further evidence that the "gruiform" buttonquails (Turnicidae) are sister to the remaining Lari, following Glareolidae (i.e., *Rhinoptilus*), then Alcidae, and last terns, gulls, and skimmers.

The combined data are also notable for what they do not support. None of the proposed relationships between shorebirds and taxa not considered charadriiform (in traditional classifications) are corroborated, with the exceptions of the "gruiform" Turnicidae and Pedionomidae. Bustards (Otididae) are not near Burhinidae, in particular, nor plovers, in general. The sister of the rails is not to be found among Jacanidae or indeed any other shorebird taxon. Ibises (Threskiornithidae) do not "link" Charadriiformes and Gruiformes. A sister relationship between sandgrouse (Pteroclidae) and Charadriiformes is also not found.

All three nuclear loci, *ADH5*, *GPD3-5*, and *FGB7*, analyzed independently or in combination yield virtually identical phylogenetic reconstructions (not shown). Individually, there is moderate conflict among the nuclear loci with respect to the position of Burhinidae. *FGB7 *supports the placement of *Burhinus *as sister to Lari plus Scolopaci with 75% bootstrap in ML analysis, but weak support (57%) in MP analysis. *ADH5 *does not resolve the position of *Burhinus *with respect to other charadriiform lineages, while *GPD3-5 *strongly supports it as sister to Charadrii, which is also the result of the combined analysis. All three data sets, individually or combined, support the Egyptian Plover (*Pluvianus aegyptius*) as sister to plovers in ML and MP analyses, rather than to the other glareolid, *Rhinoptilus*.

Our ~3.5 kb of mitochondrial data alone recovered precisely the same higher-level charadriiform relationships as did nuclear data alone, but with no bootstrap support to vouchsafe either the positions or monophyly of the three major clades. However, > 12 kb of mtDNA does recover bootstrap support all these clades [2]. Conclusions supported by our smaller mtDNA data set include the non-monophyly of Glareolidae, with *Pluvianus *nearer to Charadrii than to Lari. Alone, our mtDNA data supported *Burhinus *and *Pluvianus *as sister taxa (albeit with no bootstrap support), a relationship that has been suggested based on morphological data. The observation that bootstrap support for this pair is higher in MP than in ML analyses might suggest that long-branch attraction may be playing a role in uniting these two relatively basal lineages.

## Discussion

High bootstrap values clearly point to the monophyly of Charadriiformes, in spite of the inclusion here of taxa that have been suggested to be ingroups or sisters of Charadriiformes. Not surprisingly, waterfowl are recovered as sister to fowl, rather than among Neoaves. Flamingos are sister to grebes and sandgrouse are sister to mesites (Mesitornithidae) among the taxa we sampled, and all are recovered as monophyletic, consistent with their interpretation as members of Metaves [7,19]. Metaves and Coronaves are hypothesized basalmost sister clades of Neoaves, whose convergent members have in some cases been classified in polyphyletic orders. Among Coronaves, the remaining putative relatives of Charadriiformes are all found to be closer to one another than to Charadriiformes. Ibises are sister to spoonbills, and they in turn are sister to a clade of rails plus cranes. Bustards, too, are among this group. Somewhat ironically, this begs the question of what is the true sister of Charadriiformes, but the answer is not forthcoming from the present data set. Ongoing analysis of a larger sample of loci suggests that Charadriiformes are sister to all other coronavian waterbirds (not shown).

Other recent DNA studies of intraordinal charadriiform phylogeny are in agreement with the results presented here, lending credibility to the phylogenetic signal present in our data and *vice versa*. Two other nuclear loci, *RAG-1 *and *myo-2 *[3,6] and nearly complete mitochondrial genomes [2] yield trees of nearly perfect congruence to those of this analysis to the extent that taxa overlap. In particular, 1) Charadriiformes are comprised of three suborders, Lari, Scolopaci, and their sister Charadrii, 2) Alcidae is nested well within the Lari rather than basal among Charadriiformes as was suggested on morphological criteria [4,5,27], 3) Turnicidae are recovered as Charadriiformes, sister to Lari. When analyzed alone, *myo-2 *produced some conflicts with our data [6]. Specifically, our data do not corroborate a MP recovery of 1) a sistership between Alcidae and Glareolidae to the exclusion of other Lari, 2) a sistership of *Rynchops *and Larinae to the exclusion of Sterninae, and 3) a sistership of Jacanidae and Thinocoridae to the exclusion of Rostratulidae. These *myo-2 *results are also incongruent with those obtained from *RAG-1*. These differences are minor, and may be attributed to three causes. *myo-2 *is relatively short compared to the other introns we studied and *RAG-1 *third positions, thus there may be a higher stochastic affect on its fewer sites. This effect may be compounded by lower rates of nucleotide substitution in *myo-2 *than in the other loci. Alternatively, these could represent validly reconstructed gene trees that differ due to incomplete lineage sorting.

One recent study based solely on mtDNA *cytb *produced markedly contrasting results that we consider problematic[28]. The authors analyzed two data sets: the "primary" data set, which included 41 complete or largely complete gene sequences, and the "expanded" data set, which included an additional 50 partial sequences. They claim to have found four major clades of Charadriiformes (Charadrii, Scolopacii, Lari, and Alci). In fact, all of their reconstructions show Lari as paraphyletic to Alci (in agreement with the present study), so the latter should not be considered distinct. The finding in their primary data set that Charadrii is paraphyletic to other Charadriiformes is not well supported and contradicts all other DNA sequence studies [2]. Even more problematic, the expanded data set recovered polyphyletic relationships of indisputably monophyletic lower-level clades, e.g., within Lari (*Sterna *sister to Glareolidae in MP tree or in a clade including Jacanidae plus Rostratulidae in the Bayesian tree) and Charadriidae (the genus *Vanellus *is included in the Scolopacidae in the MP tree). Furthermore, Stercorariidae are recovered as members of the Alcidae in the MP tree but as sister to Lari plus Alcidae in the Bayesian tree. These incongruencies receive no statistical support from bootstrap analysis, and a Lento plot showed that conflict equaled or exceeded support for most clades. Indeed, the splits having the highest support/conflict ratios were either congruent with our results and previous studies (e.g., monophyly of a clade containing *Charadrius*, *Haematopus*, and *Recurvirostra*) or represented closely related taxa (e.g., species within genera).

Spurious associations of taxa from analyses of the expanded *cytb *data set may be explained as attraction between non-overlapping 5-prime and 3-prime partial sequences (e.g., 5-prime sequence for *Sterna *and 3-prime sequence for jacanids). More insidious problems with the *cytb *locus for phylogenetic reconstruction are issues of possible substitutional saturation and base composition bias. Thomas et al. [28]report that they detected no non-stationarity of base composition, nor did they apparently assess the potential for saturation. We conducted a Chi-Square test of their primary data set that shows third positions of codons to be significantly biased (Chi-Square = 151.267077, df = 120, P = 0.028; outgroups excluded). We suggest that the difference in our results accrues from partitioning the data by codon position and potentially our exclusion of constant sites (Thomas et al. did not provide details on how they conducted the test). The disparity index test in MEGA further revealed that 10.8% of all pairwise comparisons were significantly heterogeneous across all codon positions in sequences of the primary data set (outgroups excluded). We also note that two of their jacanid sequences include a deletional frameshift, suggesting that the sequences may represent nuclear pseudogenes if they are free of errors.

Inclusion of *cytb *sequences in our own concatenated data set resulted in reduced support for most clades, even though the same phylogenetic relationships were generally recovered (not shown). Moreover, Paton & Baker [2] found that *cytb *performed more poorly than most other mitochondrial genes in recovering charadriiform phylogeny. In contrast, they found that *12S rDNA *alone recovered 12 of 19 nodes and *16S rDNA *recovered 8 of 19 nodes in the combined mitochondrial tree. We found that the *rDNA*s together were able to recover 15 of 17 nodes listed by Paton and Baker [2], while the difference results only from incomplete taxonomic overlap between our study and theirs.

DNA-DNA hybridization [21] yielded a similar topology for Charadriiformes as a whole, with exceptions as already noted by Paton et al. [3] on the monophyly of Thinocoridae plus Rostratulidae plus Jacanidae, the inclusion or direct sistership of Pteroclidae, and the exclusion of Turnicidae. Unfortunately, Sibley & Ahlquist did not publish a record of what subsets of taxa were used in pairwise comparisons to produce their supertrees.

Supertree analysis [23] based on morphology, DNA-DNA hybridization, *RAG-1*, *myo-2*, and *cytb *is in near perfect agreement with the combined analysis of the 4 loci here, with the one exception of our recovery of Charadriidae as paraphyletic to Haematopodidae and Recurvirostridae that was also recovered in the study of *myo-2*. The supertree study did not include Turnicidae, and its authors asserted that further work was needed to establish their affinities[23]. Additional evidence for the placement of Turnicidae within Charadriiformes provided by this study, Fain & Houde [7], and Paton & Baker [2] suggests this question is now irrefragably resolved.

*myo-2*, *FGB7*, *ADH5*, and *GPD3-5*, whether analyzed separately or combined, all recovered Charadriidae as paraphyletic to Haematopodidae plus Recurvirostridae [6]. *RAG-1 *produced results that are consistent with these, but sequence was unavailable for the genus *Pluvialis*, which was essential in demonstrating charadriid paraphyly in all of the other data sets. Reciprocal monophyly of Charadriidae and Haematopodidae plus Recurvirostridae in the Thomas et al. supertree is clearly biased by the relative abundance of taxa for which only morphological data were available in that study. With bootstrap support as high as 100% in our complete molecular data set, the paraphyly of Charadriidae is a hypothesis that warrants serious attention.

The position of skimmers has not been consistently resolved in previous studies. Skimmers are traditionally placed in their own family Rynchopidae, sister to both terns and gulls (Laridae: Sterninae and Larinae, resp.). Ericson et al. [6] recovered this relationship with little or no support, but Paton et al. [3] obtained some support (Bayesian posterior probability 87%) for the sistership of skimmers to gulls alone. In contrast, our nuclear data strongly support a sister relationship of skimmers to terns alone (MP bootstrap = 100%), and this result is robust to combined analysis with *RAG-1 *and *myo-2*.

The Egyptian Plover (*Pluvianus aegyptius*) is traditionally placed within the family Glareolidae, although it has always been acknowledged as being atypical of the family. Some authors have suggested that the Egyptian Plover is most closely related to stone curlew (Burhinidae) based on osteological characters [4,5]. Dove [29] further noted it is atypical of the Glareolidae in microscopic feather characters. It shares with *Rhinoptilus cinctus *the peculiar habit of incubating its eggs by burying them in sandy soil [30]. At times, it has been given its own family rank, Pluvianidae [21,30].

Despite its distinctiveness as an adult, Lowe concluded that the Egyptian Plover was "obviously an advanced courser" on the basis of its natal plumage [31]. Jehl concluded instead that " [t]he color pattern of the Egyptian Plover chick is plover-like [32]" as is the relative length of the tarsus [to wing] and lack of pectination of middle toe of the adult. He further asserted that its tarsal scutellation and relative lack of flattening of anterior toes was intermediate between those of glareolids and "charadriines."

Others have questioned the placement of the Egyptian Plover in the Glareolidae, and it has even been afforded its own familial status by some [33]. Strauch [4], Mickevich & Parenti [34], and Chu [5] each studied variants of the same osteological data set using different methods of analysis. All concluded that the Egyptian Plover is sister to stone curlew, although they differed on whether the Egyptian Plover-stone curlew clade is closest to gulls, coursers, or to plovers. Thomas et al.'s supertree study positioned Egyptian Plover outside of the Glareolidae, as sister to *Burhinus*, on the basis of morphological characters alone. The authors lamented that, "Morphological studies have failed to resolve the position of Glareolidae, placing the family in a large polytomy with all other major groups except Alcinae and the sandpipers and allies[23]." We surmise that these earlier difficulties may have arisen from the polyphyletic nature of the Glareolidae with Egyptian Plover included.

Our data provide no unequivocal evidence in support of a special relationship between the Egyptian Plover and stone curlews. Both are fairly basal among Charadrii; thus, it is possible that the morphological characters they share are merely symplesiomorphies. The nuclear data obtained here, analyzed separately and in combination, strongly suggest that the Egyptian Plover is sister to a clade of plovers and allies. This phylogenetic position is consistent with previous proposals that it merits family status as Pluvianidae [33]. We were unable to compare Egyptian Plover to sheath bills, Crab Plover, and Ibisbill, and no one else has made these direct comparisons either. It is conceivable that these missing taxa could affect the grouping of Egyptian Plover with these or even other taxa among Charadriiformes.

While none of the aforementioned DNA sequence or hybridization studies included the Egyptian Plover, they all agree that the Glareolidae is sister to jaegers, auks, and gulls plus terns plus skimmers. The Glareolidae traditionally includes two subfamilies, the coursers (Cursoriinae) and pratincoles (Glareolinae), which are sufficiently distinct to cause some to question their monophyly [10]. Coursers have slender bills, long legs, and stubby tails, whereas pratincoles are swallow-like with short wide bills, short legs, and gracefully long pointed wings and tail. Despite these anatomical disparities, the monophyly of coursers plus pratincoles was strongly supported by the one DNA sequence study that included representatives of both [3]. Our treatment of the Egyptian Plover in no way challenges that result.

**Figure 4:**
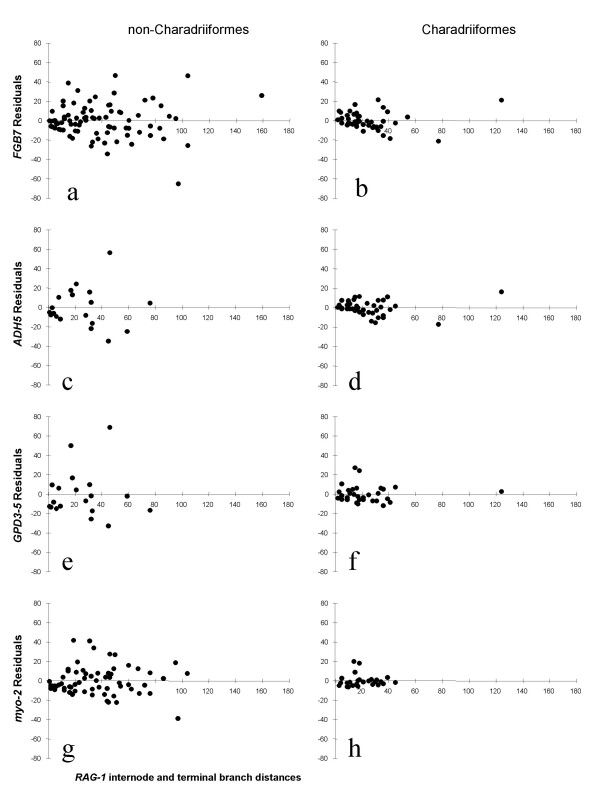
**Residual plots**. Residual plots of internodal distances of each of four nuclear loci obtained from regression against *RAG-1 *internodal distances (independent variable) on MP tree reconstructed from combined data sets of 5 nuclear loci. (a, b), *FGB7*; (c, d), *ADH5*; (e, f), *GPD3-5*; and (g, h), *myo-2*. Note the higher variance of residuals in non-Charadriiformes (left panel) than Charadriiformes (right panel), indicating better correlation of estimates of internode lengths in the latter across all loci.

It is remarkable that the phylogeny of Charadriiformes is so consistently and congruently recovered from a variety of loci, when in general, higher level relationships among Aves are notoriously difficult to resolve [35]. Even though rates of evolution differ dramatically between different lineages of Charadriiformes, it appears that these rates are lineage specific and independent of locus. For example, all loci are congruent in recovering buttonquails on a long branch, yet short branches among sandpipers. Thus, we reasoned that there might be lesser conflict between data sets within Charadriiformes than among other birds. To quantify this, we constructed a MP tree using a supermatrix data set of *FGB7*, *ADH5*, *GPD3-5*, *RAG-1*, and *myo-2*. Distances were then determined for all internodes and terminals for each of the loci individually, and these were regressed against one another using the *RAG-1 *distances as the independent variable. The residuals obtained from regressing each locus against those of *RAG-1 *were next segregated and plotted on the basis of whether the branches were within (fig. 4, right panel) or outside (fig. 4, left panel) the Charadriiformes clade.

The sample variance of the residuals for Charadriiformes was in every case significantly smaller than for non-Charadriiformes as determined by two-tailed sample variance ratio test (Table 4). Similarly, the sample means of the absolute values of the residuals (absolute because residuals are both positive and negative) are significantly smaller for Charadriiformes than non-Charadriiformes as determined using the two-tailed normal approximation to the Mann-Whitney Test [36]. The lesser mean and variance of residuals reflect the relatively higher correlation of estimation of internodal branch lengths between data sets within Charadriiformes, not shorter distances (although note *r*^2 ^is smaller for Charadriiformes than non-Charadriiformes for *myo-2*). We interpret these statistics to indicate that there is significantly less conflict in the nuclear locus data sets among Charadriiformes than among non-Charadriiformes.

**Table 4:** Descriptive statistics of internodal regression analysis

		Residuals					
		
Regression	*r*^2^	sample variance	*F *ratio and significance	n	range	mean of absolute residuals	Z value and significance
**b-fib7 X RAG-1**							
non-Charadriiformes	0.56	308.7	3.811	82	111.9	12.7	2.853
Charadriiformes	0.77	81.0	P < 0.001	45	42.9	6.7	P < 0.005
**ADH-5 X RAG-1**							
non-Charadriiformes	0.29	426.0	8.192	19	91.4	15.5	3.779
Charadriiformes	0.67	52.0	P < 0.001	43	33.3	5.5	P < 0.001
**GPD3-5 X RAG-1**							
non-Charadriiformes	0.13	605.5	7.915	19	101.5	17.3	3.468
Charadriiformes	0.42	76.5	P < 0.001	32	39.0	6.4	P < 0.001
**myo-2 X RAG-1**							
non-Charadriiformes	0.31	210.7	5.077	66	80.6	11.0	4.706
Charadriiformes	0.10	41.5	P < 0.001	27	26.2	4.2	P < 0.001

It is important to note that the values used to produce Figure 4 represent globally optimized internodes rather than measured pairwise distances between taxa. In this regard we have avoided issues of autocorrelation of pairwise distances along shared branches. Furthermore, the lengths of the internodes are not correlated with their depth in the tree. This can be appreciated by the broad overlap of data on the horizontal axes between shorebirds (fig. 4, right panel) and non-shorebirds (fig. 4, left panel) in all but the *myo-2 *locus.

If character conflict among data sets is reduced in Charadriiformes, then it might make the reconstruction of their phylogeny more tractable than those of many other birds. After all, the extent to which various loci yield congruent phylogenetic reconstructions of Charadriiformes is presumably reflective of the degree to which each is informative individually. The reason(s) that certain clades should be more readily recovered than others in phylogenetic analysis is not always self-evident. The age of lineages does not appear to be a primary factor because the most ancient avian divergences, such as between paleognaths and neognaths or between Galloanserae and Neoaves, are among those recovered with the greatest reproducibility, irrespective of data set. The shortness of internodes has been implicated as an impediment to phylogenetic resolution at least in the extreme case of "explosive radiations" [35]. While it might seem intuitive that internode length should be a factor, our residual plots suggest otherwise, at least in the case of shorebirds. This is because *RAG-1 *has been shown to be clock-like in shorebirds [3] and there is broad overlap between internode distances (i.e., not pairwise distances) within Charadriiformes versus non-Charadriiformes.

Hypothetically, high support might be obtained even for congruent gene-phylogenies that yield vastly different length estimates of the same branches. For instance, despite relatively strong support for the rostratulid-jacanid clade, *FGB7 *shows a very short internode to these taxa; but, this is neither the case for *ADH5 *nor *GPD3-5*. On the contrary, it seems in this case that there is lesser support for parts of the tree where different loci yield more conflict in estimates of branch length. This is intuitively satisfying, though the cause for conflicts in individual gene trees remains obscure. Conflict may be a result of sampling error or of truly different gene genealogies. An argument for sampling error might be made from the observation that the substitution rates of "neutral" partitions (i.e., introns and third positions) appear to be correlated with support. Specifically, *myo-2 *had the lowest substitution rate relative to other loci and recovered the most nodes inconsistent with the combined tree, as well as having lower bootstrap support values. Future studies might profitably consider whether clade-specific differences such as genomic composition might adversely affect phylogenetic analysis at multiple loci in a lineage specific fashion.

## Conclusion

Charadriiformes represent a monophyletic group that neither includes nor is sister to waterfowl, flamingos, bustards, sandgrouse, ibises, cranes or rails. Exactly what their sister relationships are remain obscure. Charadriiformes consist of three monophyletic suborders Lari (i.e., Laridae [including Sternidae and Rynchopidae], Stercorariidae, Alcidae, Glareolidae, Turnicidae, and presumably Dromadidae), Scolopaci (i.e., Scolopacidae [including Phalaropidae], Jacanidae, Rostratulidae, Thinocoridae, Pedionomidae), and Charadrii (i.e., Burhinidae, Chionididae, Charadriidae, [including Haematopodidae, and Recurvirostridae], and presumably Ibidorhynchidae). Skimmers are most likely sister to terns alone, and plovers may be paraphyletic with respect to oystercatchers and stilts. The Egyptian Plover is not a member of the Glareolidae, but is instead relatively basal among Charadrii.

## Methods

Taxa sampled and GenBank accession numbers are listed in Table 1. A galliform (*Callipepla gambelii*) and an anseriform (*Anseranas semipalmata*) were designated as the root, as they represent the Galloanserae, widely accepted as the monophyletic sister clade to Neoaves in which all other taxa in this study are included.

Sequences of mitochondrial-encoded *12S rDNA*, *tRNA-Valine*, and *16S rDNA*, and three nuclear loci; *alcohol dehydrogenase-I intron 5*, *glyceraldehyde-3-phosphate dehydrogenase exons 4–5 and introns 3–5*, and *β-fibrinogen intron 7 *were amplified from genomic DNA using primers as described [22,24,37,38]. PCR reaction conditions were: 35 cycles of 94°C denature, 55°-60°C annealing (depending on primer pair), 72°C extension, for one minute each step. Amplicons were purified by agarose gel electrophoresis and QIAquick gel extraction kit (Qiagen, Inc., Valencia, CA) according to manufacturer's instructions. Cycle sequencing was performed using the above primers, according to manufacturer's instructions using BigDye v3.1 and read on an ABI 3100 DNA sequencer.

Sequences were aligned using Se-Al v2.0a11[39]. Alignments for mitochondrial RNA genes followed secondary structures as templates to attempt to maximize homologous positions [22,40,41]. Alignments for the nuclear introns were generally straightforward, but were algorithmically aligned with ClustalX 1.8 [42] and MUSCLE [43] and adjusted by hand. Differences corresponded to regions where a multiple alignments could justifiably be produced; these regions of ambiguous alignment were removed prior to the phylogenetic analysis.

Phylogenetic analyses were conducted by equally-weighted maximum parsimony, with gaps treated as missing data, using PAUP*4.0b10 [44]. Maximum likelihood analyses were performed using PHYML v2.44 [45], and mixed-model analysis was implemented in MrBayes 3.0. Compositional stationarity was explored using the Chi-Square test in PAUP, and in some cases further investigated by the pairwise disparity index analysis in MEGA 3.1. Trees for selection of nucleotide substitution models were obtained in PAUP* from neighbor-joining analyses using an F84 model. The best-fitting substitution model for the ML analyses was chosen by hierarchical likelihood ratio tests and the Akaike information criterion implemented in ModelTest v3.06 [46]. For these data, where the two methods differed, we chose the less parameter-rich model. In the case of *ADH5*, we selected a "compromise" model by relaxing the assumption of equal base frequencies for the Tamura-Nei model. This appeared justified because the selection of equal base frequencies by hLRT is likely a consequence of combining short conserved segments of flanking exon sequence with the intron. Table 3 shows models and associated parameter values used in tree-searches with PHYML. The best tree obtained was submitted to PAUP for a further tree-bisection-reconnection search. Statistical support for the resulting phylogenies was assayed by conducting 500 bootstrap pseudoreplicate searches, also completed in PHYML. Mixed-model Bayesian analysis also employed locus-specific models comparable to those chosen for ML analyses of individual genes.

## List of Abbreviations

*12S rRNA *– *small subunit ribosomal ribonucleic acid*

*16S rRNA *– *large subunit ribosomal ribonucleic acid*

ABI – Applied Biosystems Incorporated

*ADH-I *– alcohol dehydrogenase-1

*ADH5 *– alcohol dehydrogenase-1 intron 5

AIC – Akaike Information Criterion

*FGB7 *– *β-fibrinogen intron 7*

bp – base pairs

*cytb *– *cytochrome-b*

+G – gamma distributed

GAPD-H – glyceraldehyde-3-phosphate dehydrogenase

*GPD3-5 *– *glyceraldehyde-3-phosphate dehydrogenase exons 4–5 and introns 3–5*

GTR – general time reversible substitution model

HKY – Hasegawa, Kishino, and Yano 85 substitution model

hLRT – hierarchical likelihood ratio test

+I – invariable proportion of sites

kb – kilobases

ML – maximum likelihood

MP – maximum parsimony

mtDNA – mitochondrial deoxyribonucleic acid

*myo-2 *– *myoglobin intron 2*

PCR – polymerase chain reaction

PI – parsimony informative

Pinv – proportion of invariant sites

*RAG-1 *– *recombination activating gene*

rDNA – ribosomal deoxyribonucleic acid

TNef – Tamura Nei equal frequencies substitution model

tRNA – transfer ribonucleic acid

TVM – transversional model

## Authors' contributions

MGF performed the sequencing. Both MGF and PH performed analyses and cooperatively wrote the manuscript. Both authors read and approved the final manuscript.

## References

[B1] Van Tuinen M, Waterhouse D, Dyke GJ (2004). Avian molecular systematics on the rebound: a fresh look at modern shorebird phylogenetic relationships.. J Avian Biol.

[B2] Paton TA, Baker AJ (2006). Sequences from 14 mitochondrial genes provide a well-supported phylogeny of Charadriiform birds congruent with the nuclear RAG-1 tree.. Mol Phylogenet Evol.

[B3] Paton TA, Baker AJ, Groth JG, Barrowclough GF (2003). RAG-1 sequences resolve phylogenetic relationships within Charadriiform birds.. Mol Phylogenet Evol.

[B4] Strauch JG (1978). The phylogeny of Charadriiformes (Aves): a new estimate using the method of character compatibility analysis.. Trans Zool Soc London.

[B5] Chu PC (1995). Phylogenetic reanalysis of Strauch’s osteological data set for the Charadriiformes.. Condor.

[B6] Ericson PGP, Envall I, Irestedt M, Norman JA (2003). Inter-familial relationships of the shorebirds (Aves: Charadriiformes) based on nuclear DNA sequence data.. BMC Evol Biol.

[B7] Fain MG, Houde P (2004). Parallel radiations in the primary clades of birds.. Evolution.

[B8] Olson SL, Feduccia A (1980). Relationships and evolution of flamingos (Aves: Phoenicopteridae).. Smithsonian Contrib Zool.

[B9] Olson SL, Feduccia A (1980). Presbyornis and the origin of the Anseriformes (Aves: Charadriimorphae).. Smithsonian Contrib Zool.

[B10] Olson SL (1985). The fossil record of birds.. Avian Biology.

[B11] Feduccia A (1996). The Origin and Evolution of Birds..

[B12] Olson SL (1979). Multiple origins of the Ciconiiformes.. Proc Colonial Waterbird Group.

[B13] Sibley CG, Ahlquist JE, deBenedictis P (1993). The phylogenetic relationships of the rails, based on DNA comparisons.. J Yamashina Inst Ornithol.

[B14] Groth JG, Barrowclough GF (1999). Basal divergences in birds and the phylogenetic utility of the nuclear RAG-1 gene.. Mol Phylogenet Evol.

[B15] Van Tuinen M, Sibley CG, Hedges SB (2000). The early history of modern birds inferred from DNA sequences of nuclear and mitochondrial ribosomal genes.. Mol Biol Evol.

[B16] Chubb AL (2004). 2004 New nuclear evidence for the oldest divergence among neognath birds: the phylogenetic utility of ZENK.. Mol Phylogenet Evol.

[B17] Van Tuinen M, Butvill DB, Kirsch JAW, Hedges SB (2001). Convergence and divergence in the evolution of aquatic birds.. Proc R Soc Lond B.

[B18] Mayr G (2004). Morphological evidence for sister group relationship between flamingos (Aves: Phoenicopteridae) and grebes (Podicipedidae).. Zool J Linn Soc.

[B19] Ericson PGP, Anderson CL, Britton T, Elzanowski A, Johansson US, Källersjö M, Ohlson JI, Parsons TJ, Zuccon D, Mayr G (2006). Diversification of Neoaves: integration of molecular sequence data and fossils.. Biol Lett.

[B20] Olson SL, Steadman DW (1981). The relationships of the Pedionomidae (Aves: Charadriiformes).. Smithsonian Contrib Zool.

[B21] Sibley CG, Ahlquist JE (1990). Phylogeny and Classification of Birds: a study in molecular evolution..

[B22] Fain MG, Krajewski C, Houde P (2007). Phylogeny of "core-Gruiformes" (Aves) and resolution of the Limpkin-Sungrebe problem.. Mol Phylogenet Evol.

[B23] Thomas GH, Wills MA, Székely T (2004). A supertree approach to shorebird phylogeny.. BMC Evol Biol.

[B24] Prychitko TM, Moore WS (1997). The utility of DNA sequences of an intron from the ß-fibrinogen gene in phylogenetic analysis of woodpeckers (Aves: Picidae).. Mol Phylogenet Evol.

[B25] Prychitko TM, Moore WS (2000). Comparative evolution of the mitochondrial cytochrome b gene and nuclear beta-Fibrinogen intron 7 in woodpeckers.. Mol Biol Evol.

[B26] Prychitko TM, Moore WS (2003). Alignment and phylogenetic analysis of beta-fibrinogen intron 7 sequences among avian orders reveal conserved regions within the intron.. Mol Biol Evol.

[B27] Björklund M (1994). Phylogenetic relationships among Charadriiformes: reanalysis of previous data.. Auk.

[B28] Thomas GH, Wills MA, Székely T (2004). Phylogeny of shorebirds, gulls, and alcids (Aves: Charadrii) from the cytochrome-b gene: parsimony, Bayesian inference, minimum evolution, and quartet puzzling.. Mol Phylogenet Evol.

[B29] Dove CJ (2000). A descriptive and phylogenetic analysis of plumulaceous feather characters in Charadriiformes.. Ornithological Monographs.

[B30] Maclean GL, del Hoyo J, Elliott A and Sargatal J (1996). Family Glareolidae (coursers and pratincoles).. Handbook of Birds of the World, Vol 3 Hoatzin to Auks.

[B31] Lowe PR (1931). An anatomical review of the "waders" (Telmatomorphae), with special reference to the families, subfamilies, and genera within the suborders Limicolae, Grui-Limicolae, and Lari-Limicolae.. Ibis.

[B32] Jehl JR (1968). Relationships in the Charadrii (shorebirds): a taxonomic study based on color patterns of the downy young.. San Diego Society of Natural History, Memoir.

[B33] Judin KA (1965). Phylogeny and classification of the Charadriiformes [in Russian].. Fauna USSR, Aves, Ser1,2 (1), ns 91Acad of Sci.

[B34] Mickevich MF, Parenti LR (1980). [Review of] The phylogeny of the Charadriiformes (Aves): A new estimate using the method of character compatibility analysis.. Syst Zool.

[B35] Poe S, Chubb AL (2004). Birds in a bush: five genes indicate explosive evolution of avian orders.. Evolution.

[B36] Zar JH (1974). Biostatistical Analysis..

[B37] Kocher TD, Thomas WK, Meyer A, Edwards SV, Pääbo S, Villablanca FX, Wilson AC (1989). Dynamics of mitochondrial DNA evolution in animals: Amplification and sequencing with conserved primers.. Proc Natl Acad Sci USA.

[B38] Sorenson MD, Ast JC, Dimcheff DE, Yuri T, Mindell DP (1999). Primers for a PCR-based approach to mitochondrial genome sequencing in birds and other vertebrates.. Mol Phylogenet Evol.

[B39] Rambaut A Se-Al: Sequence Alignment Editor.. http://evolve.zoo.ox.ac.uk.

[B40] Desjardins P, Morais R (1990). Sequence and gene organization of the chicken mitochondrial genome: a novel gene order in higher vertebrates.. J Mol Biol.

[B41] Houde P, Cooper A, Leslie E, Strand AE, Montaño GA, Mindell DP (1997). Phylogeny and evolution of 12S rDNA in Gruiformes (Aves).. Avian Molecular Evolution and Systematics.

[B42] Thompson JD, Gibson TJ, Plewniak F, Jeanmougin F, Higgins DG (1997). The ClustalX windows interface: flexible strategies for multiple sequence alignment aided by quality analysis tools.. Nucl Acids Res.

[B43] Edgar RC (2004). MUSCLE: multiple sequence alignment with high accuracy and high throughput.. Nucl Acids Res.

[B44] Swofford DL PAUP*: phylogenetic analysis using parsimony (* and other methods), version 4.0b10..

[B45] Guindon S, Gascuel O (2003). A simple, fast, and accurate algorithm to estimate large phylogenies by maximum likelihood.. Syst Biol.

[B46] Posada D, Crandall KA (1998). Modeltest: testing the model of DNA substitution.. Bioinformatics.

